# Cruciate-Retaining Total Knee Arthroplasty: Current Concepts Review

**DOI:** 10.7759/cureus.43813

**Published:** 2023-08-20

**Authors:** Kristine Yang, Garrett Sohn, Senthil Sambandam

**Affiliations:** 1 Orthopedics, University of Texas Southwestern Medical Center, Dallas, USA

**Keywords:** cruciate-retaining, cruciate-retaining tka, total knee arthroplasty implants, total knee arthroplasty, cr tka

## Abstract

Posterior cruciate-retaining (CR) total knee arthroplasty for osteoarthritis of the knee is a popular implant choice. At present, there is no consensus on whether sacrifice or retention of the posterior cruciate ligament (PCL) offers superior outcomes. This review explores the current literature available on CR total knee arthroplasty (TKA). PubMed was searched by keyword to find relevant articles for inclusion. Additional sources came from article references and joint registry reports. CR design knees have distinct kinematic gait patterns from posterior-stabilizing (PS) knees and exhibit paradoxical anterior femoral movement with less femoral rollback. While CR implants offer less flexion than PS designs, the difference is not clinically detectable as clinical scores are similar in the short and long term. CR implants have better long-term survival compared to PS knees, likely due to lower risk of aseptic loosening. CR total knee arthroplasties also have shorter operating times and lower risk of peri-prosthetic fractures. Because the CR implant is unconstrained, there may be an increased risk of instability compared to PS designs, but the literature is mixed. Overall, the current literature supports the continued use of CR TKAs due to their lower risk of complications, durability, and demonstrated equivalence in function to posterior-substituting models.

## Introduction and background

Osteoarthritis (OA) is one of the leading causes of disability worldwide and its true burden on population health is likely underestimated. Approximately 3.8% of the global population is estimated to be affected by knee OA [1], and the knee is the most commonly replaced joint [2]. Primary total knee arthroplasty (TKA) offers definitive surgical management for refractory pain or functional limitation due to OA of the knee, amongst other painful knee conditions such as rheumatoid arthritis. The number of primary TKAs is projected to increase in the next 20 years due to age- and obesity-associated increase in both prevalence and incidence of OA [2]. 

Various joint replacement systems are available for TKA. Cruciate-retaining (CR) and posterior-stabilizing (PS) implants are the most widely utilized. In the United States. Surgeons prefer PS implants over CR, although CR implant designs are growing in popularity [3]. In European countries, the CR model is more widely used [4-8]. Both CR and PS models offer unique advantages and drawbacks, but there remains no consensus on which offers superior outcomes. This study aims to review the scope of current literature on CR implants for TKA, specifically focusing on kinematics, functional outcomes, survival, and complications.

Methods

**Figure 1:**
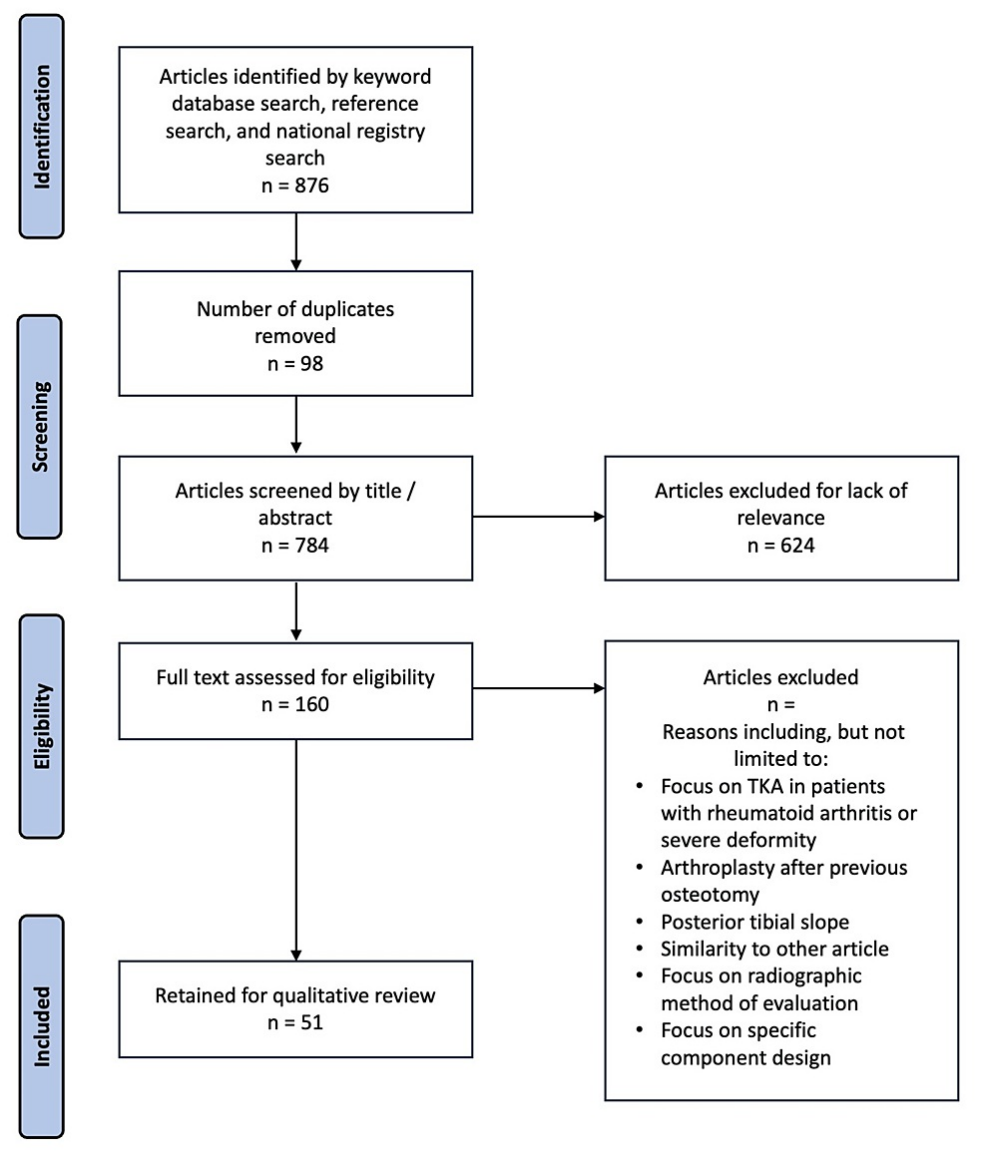
Article selection process. TKA: total knee arthroplasty

For each section of the article, a PubMed search utilizing relevant keywords was conducted. Keywords used were "native knee flexion kinematics" (n = 308), "CR PS total knee kinematics" (n = 80), "CR PS knee outcomes" (n = 107), "CR knee survival" (n = 51), "PS knee survival" (n = 77), "CR PS knee flexion" (n = 97), "CR TKA revision" (n = 61), "CR TKA instability" (n = 89). Six additional references were obtained from 2022 national registry reports. Articles that compared CR and PS TKAs in terms of any metric were of special interest. Two independent reviewers screened articles based on titles. Those that were deemed suitable for abstract review were compiled into a list that was independently screened by two reviewers for retention or exclusion based on relevance. A total of 51 articles were included in the review (Figure 1). Articles were excluded if they were not about CR TKAs, were about TKAs for treatment of conditions other than knee OA, TKAs on patients who had undergone previous knee surgeries, focus on posterior tibial slope, focus on soft tissue deformities, and focus on component-specific design such as polyethylene design. 

## Review

The native knee

Structures of the Knee

Articular surfaces of the native knee involve medial and lateral femoral condyles, which articulate with the respective condyles of the tibial plateau and are supported by the surrounding menisci. The ligamentous structures, which include the anterior cruciate ligament, medial collateral ligament, lateral collateral ligament, and posterior cruciate ligament (PCL) serve to provide tension and stability to the knee joint during movement.

The PCL is an intra-articular structure of the knee joint with its own synovial sheath. It is composed of the anterolateral bundle (ALB) and the posteromedial bundle (PMB). The ALB originates anteriorly compared to the PCL on the medial epicondyle of the femur and inserts anteriorly and medially to the PMB on the posterior tibia. Compared to the PMB which runs an oblique course relative to axial load on the knee joint, the ALB is situated upright. Both bundles resist posterior translation of the tibia in a codominant fashion during extension and flexion of the knee joint. During maximal extension of the knee joint, the PMB is stretched taut and acts as the dominant bundle to resist posterior tibial translation. In contrast, the ALB elongates during mid-flexion and resists posterior tibial translation while the PMB is lax. During extreme flexion, the PMB is again dominant and serves to resist posterior translation and internal rotation of the tibia. 

Kinematics

During knee flexion, the asymmetric articular surfaces of the knee along with the PCL enable femoral rollback and external rotation of the femur with respect to the tibia. The medial tibial plateau is larger and concave, while the lateral tibial plateau is convex. As a result, medial condylar contacts are more static while lateral contact points are more variable, causing a pivot about the medial condyles. From extension to 20º of flexion, condylar contact points rotate externally and posteriorly as the PMB exerts anterior and external rotary force on the tibia. During mid-flexion up to about 80º of flexion, the medial and lateral condylar contact points both translate posteriorly owing to the centrally located ALB providing anterior forces on the tibia. During flexion >90º, the lateral contact points translate posteriorly while the medial contact points remain static due to the PMB [9].

**Figure 2:**
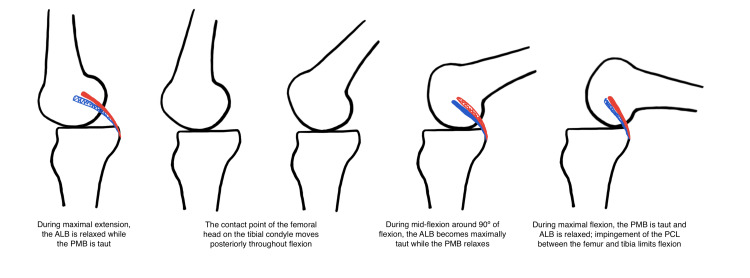
Femoral rollback with posterior cruciate ligament (PCL) components. Original illustration. Blue represents the anterolateral bundle (ALB) of the PCL, and red represents the posteromedial bundle (PMB) of the PCL.

The net effect of knee flexion is a posterior translation of the femoral head with respect to the tibia known as “femoral rollback” (Figure 2). This is opposed by the “screw home” mechanism in extension, which is an external tibial rotation with relative internal femoral rotation centered on the medial condyle that provides stability during full knee extension. Coordination between the ALB and PMB of the PCL creates constraint and stability throughout all ranges of extension and flexion. 

Kinematics after arthroplasty

Due to preservation of the native PCL, CR arthroplasty designs allow for stability throughout the flexion-extension arc, detailed above. Kinematic studies of CR designs, often in comparison to PS designs, provide understanding of the effects of PCL preservation. In addition to passive flexion, mobility patterns critical for activities of daily living such as walking gait and stair climbing are of particular interest when studying TKA kinematics.

Gait Kinematics

CR implant designs generally allow posterior translation of femorotibial condylar contact points in early flexion <30º, followed by paradoxical anterior femoral sliding from 30-60º during normal gait kinematic analysis [10-12]. In comparison to PS designs, which do not retain the native PCL, condylar contact points in CR designs are overall relatively anterior from 30-60º. The difference was most significantly exaggerated in deeper knee flexion >60º, which is not engaged during normal gait [13]. Studies comparing kinematics of CR and PS designs during normal gait report CR knees demonstrated an increased “pivot” moment about the medial compartment, as evidenced by higher lateral-to-medial rollback ratios [12-14].

Stair-Climbing Kinematics

During stair climbing, Hamai et al. found that both CR and PS designs demonstrate paradoxical posterior femoral translation during early flexion due to absence of the ACL [15]. In CR models, the PCL provided mid-flexion stability, while PS designs demonstrated risk of paradoxical anterior translation in mid-flexion. Flexion during stair-climbing was not sufficient to engage the cam-post mechanism of PS implants [15]. Fantozzi et al. found the medial condylar contact point for CR implants consistently translated posteriorly during stair climbing to a degree similar to PS designs until about 60º of flexion, upon which PS medial condylar contacts shifted significantly more posteriorly [14].

Passive Flexion Kinematics

Intra-operative studies investigating the kinematics of passive flexion in CR implants against PS implants in the same knee demonstrate different patterns of condylar contact at varying degrees of flexion. From 0-30º of flexion, studies agree both CR and PS designs demonstrate a posterior shift of the condylar contact points [10,11,13]. From 30-60º of flexion, CR knee contact points were variable, and some knees demonstrated paradoxical anterior femoral motion [10-12]. Translation of contact points during 30-60º of flexion was not observed in PS knees, in which condylar contact points remained stable [10,12]. In mid-flexion from 60-90º, CR knee contact points were significantly anterior compared to the PS knee contact points, although both knees demonstrated posterior translation [11,13]. In deep passive flexion >90º, CR knees had significantly higher rates of paradoxical anterior femoral translation than PS knees; however, not all CR knees demonstrated paradoxical anterior femur translation [10,13]. 

Femoral Rollback

A recent meta-analysis by Li et al. suggests that CR knees retain less native femoral rollback than PS knees (80% vs 90%) [16], consistent with the kinematics literature previously discussed. Yoshiya et al. report that CR implants also demonstrate more variable results in terms of kinematics, which may reflect variability of native PCL condition [10]. Exclusive lateral condylar liftoff has been noted in CR TKAs, as opposed to PS TKAs in which both medial and lateral condylar liftoff has been noted [12].

Functional outcomes

Short-Term Functional Outcomes

Most studies on CR TKA functional outcomes are limited to short-term follow-up, defined here as two to five years after primary TKA. Overall, in this period, studies suggest CR and PS TKAs offer equivalent clinical scores as assessed by the Oxford Knee Score (OKS), Knee Society Pain Score and Knee Society Functional Score (KSS) [17-22]. However, certain studies have demonstrated better clinical scores associated with PS implant designs [23-26]. The Forgotten Joint Score (FJS) is a newer, validated, and reliable metric with lower ceiling effect than the OKS and KSS and allows for differentiation among patients with positive TKA outcomes [27-29]. FJS score showed no significant difference between CR and PS TKAs overall and in all subsets of age, gender, or laterality in a study by Thippanna et al. [17], but was significantly higher for CR knees in a study by Thuysbayert et al. [28]. Based on the current literature, it appears that neither CR nor PS implants have a distinct advantage over the other on short-term follow-up.

Long-Term Functional Outcomes

Long-term functional outcomes, defined as more than five years after primary TKA, appear similar between CR and PS designs as well. A 10-year minimum case-control study by Serna-Berna et al. found no differences in clinical scores or patient satisfaction [22]. Mouttett et al. noted equivalent pre- and post-operative International Knee Society (IKS) scores at seven-year follow-up for CR and PS TKAs [21]. One study by Sando et al. found significantly better clinical scores for PS than CR models at 10-year follow-up [30], while Rajgopal et al. observed better clinical scores at 15-year follow-up for CR models [31]. Although the current data is mixed, further study on long-term functional outcomes is warranted.

Range of Motion

In general, CR TKA designs have smaller flexion arcs than PS designs, reported to be between 2.24-4.05º less (Table 1) [12,16,18,23,26,30,32,33]. Some studies have noted similar knee scores and range of motion (ROM) regardless of PCL sacrifice [24,34]. Longo et al. additionally found PS knees offer significantly better extension as compared to CR designs [25]. The disparity in ROM is generally considered clinically insignificant, as flexion differences of <5º have been found imperceptible by patients [19,34].

**Table 1:** Flexion range of motion (ROM) in cruciate-retaining (CR) and posterior-stabilized (PS) implant designs. NA: data not available

Author	CR flexion ROM (º)	PS flexion ROM (º)	Difference in flexion (CR - PS) (º)	Significance
Dennis 2003 [12]	121 ± 16	131 ± 12	NA	p<0.05
Li 2022 [16]	NA	NA	-3.2 ± 0.28	p=0.03
Maruyama 2004 [18]	122.3 ± 15	131.3 ± 13.4	NA	p<0.05
Luo 2012 [19]	NA	NA	-4.34 ± 1.16	p<0.01
Li 2014 [20]	NA	NA	-2.88 ± 2.75	p=0.04
Mouttett 2014 [21]	110	115	NA	p=0.12
Verra 2013 [23]	118.3	115.9	-2.4 ± 2.27	p=0.04
Scott 2014 [24]	124.1	125.8	NA	p=0.87
Longo 2018 [25]	115.2	119.4	NA	p<0.00001
Migliorini 2019 [26]	NA	NA	-4.05 ± 0.96	p=0.004
Sando 2015 [30]	113.5 ± 13.4	116.7 ± 12.8	NA	p=0.06
Rajgopal 2021 [31]	125.7 ± 3.8	125.1 ± 3.1	NA	p=0.07
Bercik 2013 [32]	NA	NA	-2.24 ± 0.57	p=0.009
Hajduk 2016 [33]	99.78 ± 12.75 SD	107.89 ± 11.46 SD	NA	p=0.13
Chaudhary 2008 [34]	105.9 ± 13.0	105.8 ± 13.5	0.03 ± 6.0	p>0.05

Survival 

Implant survivability is of paramount importance to both the patient and the treating surgeon. Short-term TKA failure is commonly due to infection, while aseptic loosening is the most common cause of long-term TKA failure [35-37]. A meta-analysis by Migliorini et al. found no significant difference in revision rate over a mean follow-up period of 3.37 years [26]. Rates of revision arthroplasty after primary TKA are equivalent for CR and PS knees at mid-term follow-up [19,20].

Current research suggests greater longevity of CR TKAs compared to PS designs [31,38-40]. Abdel et al. conducted a retrospective review of over 8,000 CR and PS TKAs performed over a 10-year period, which revealed significantly improved survival for CR compared to PS TKAs at 15-year follow-up, with survival rates of 90% and 77% respectively and a hazard ratio of 0.5 for revision favoring CR implants [39]. A recent systematic review and meta-analysis by Kanna et al. also showed greater CR than PS TKA survival with a 10-year survival odds-ratio of 2.22 and a 15-year survival odds-ratio of 2.48 [38]. Ten-year national registry information additionally supports greater 10-year survival rates of CR designs (Table 2) [3,5,6,8,41].

**Table 2:** 2022 registry reports comparing 10-year revision for cruciate-retaining (CR) and posterior-stabilizing (PS) implants. NA: data not available.

Registry	10-year CR revision	10-year PS revision	10 year hazard ratio (CR vs PS)	Qualifiers	p-value
American Joint Replacement Registry 2022 [3]	NA	NA	0.776	NA	p<0.0001
Australian Orthopaedic Association National Joint Registry 2022 [5]	5.0 (4.7 - 5.3)	5.1 (4.8 - 5.5)	NA	Genesis II femoral / Genesis II tibial	NA
	4.5 (3.8 - 5.2)	5.8 (5.3 - 6.3)	NA	Legion Oxinium femoral / Genesis II tibial	NA
	5.2 (4.4 - 6.3)	4.3 (3.7 - 5.1)	NA	Legion femoral / Genesis II tibial	NA
	3.1 (2.8 - 3.9)	4.9 (4.4 - 5.5)	NA	Nexgen femoral / Nexgen tibial	NA
	3.1 (3.0 - 3.3)	5.0 (4.8 - 5.3)	NA	Nexgen flex femoral / Nexgen tibial	NA
	5.1 (4.5 - 5.7)	5.4 (4.8 - 6.0)	NA	PFC sigma femoral / MBT tibial	NA
	3.5 (3.3 - 3.8)	4.7 (4.2 - 5.3)	NA	PFC sigma femoral / PFC sigma tibial	NA
	3.7 (3.6 - 3.9)	5.5 (5.1 - 6.0)	NA	Triathlon femoral / Triathlon tibial	NA
	4.9 (4.6 - 5.3)	7.3 (6.5 - 8.2)	NA	Vanguard femoral / Vanguard tibial	NA
UK National Joint Registry 2022 [6]	2.83 (2.78 - 2.87)	3.83 (3.74 - 3.92)	NA	Fixed	NA
	4.04 (3.83 - 4.26)	4.14 (3.78 - 4.54)	NA	Mobile	NA
New Zealand Joint Replacement Registry 2022 [7]	0.41 (0.39 - 0.42) [rate/100 component years]	0.59 (0.56 - 0.62) [rate/100 component years]	NA	NA	NA
Swedish Joint Replacement Registry 2022 [8]	NA	NA	1.03 (0.83 - 1.27)	NA	p=0.8
Canadian Joint Replacement Registry 2022 [41]	NA	NA	0.81 (0.75 - 0.88)	No patella	p=0.0001
	NA	NA	0.81 (0.76 - 0.87)	Patellar resurfaced	p=0.0001
	NA	NA	0.83 (0.79 - 0.88)	Fixed-bearing	p<0.0001
	NA	NA	0.43 (0.31 - 0.60)	Mobile-bearing	p<0.0001

Aseptic loosening has been found to occur significantly less frequently in CR TKAs compared to PS TKAs, while infection rates are not significantly different between the two arthroplasty types [37,38]. At 13-year follow-up, 93% of revisions for PS designs were due to loosening, and males were at significantly higher risk [37]. Greater frequency of aseptic loosening in PS knees may explain better long-term survival of CR TKAs compared to PS TKAs. While certain studies do not support CR design superiority regarding longevity [30], more suggest at least equivalent long-term survival outcomes (Table 3) [21,22,30,31,38,39].

**Table 3:** Articles reviewed comparing long-term survival between cruciate-retaining (CR) and posterior-stabilizing (PS) implants. TKA: total knee arthroplasty, ROM: range of motion

Author	Type (Institution / Registry / Meta)	Follow-up time (years)	#Knees	Findings
Mouttet 2014 [21]	Institution	Mean 4.5 (range 1-9.6)	114	No significant difference found in post-operative scores between CR and PS groups. With revision for any reason as the endpoint, seven-year overall implant survival was 94.8% in the CR group and 97.5% in the PS group. No significant difference in survival was found between the two designs.
Serna-Berna 2018 [22]	Institution	Minimum 10 years	268 CR / 211 PS	No significant difference found in functional scores, range of motion, patient-related scores, or patient satisfaction. Between the 5-year and last postoperative follow-up, there were significant decreases of all clinical scores in both groups. Complication rate and implant survival were similar between groups.
Sando 2015 [30]	Institution	Mean 12.3 (range 10.2-14.4)	143 CR / 271 PS	Post-operative clinical scores and ROM were significantly better for the PS cohort.
Rajgopal 2021 [31]	Institution	Minimum of 15 (range 15-21)	204 CR / 124 PS	CR and PS designs showed good long-term survivorship and similar rates of all-cause revision for patients <55 years. CR design showed slightly better longevity, though the difference was not statistically significant. Deep infection and aseptic loosening were the most common causes of revision.
Kanna 2023 [38]	Meta-analysis	Minimum 10 years	14189 CR / 6650 PS	CR designs had significantly better survival 10 years post-operatively than PS designs, but the complication rate was not different. Outcomes also were not significantly different between the two designs.
Abdel 2011 [39]	Institution	Median 10.2 (range 1 day to 20.4 years)	5389 CR / 2728 PS	Survival at 15 years was 90% for CR designs, compared with 77% for PS designs. The greater survival of CR and PS knees was more pronounced in knees with severe preoperative deformity at 90% and 75% respectively. After adjustment for age, sex, preoperative diagnosis, and deformity, risk of revision remained significantly lower in CR TKAs compared to PS designs.

Knee arthroplasty complications

Fractures

Complications for TKA include fractures, pain, and unplanned conversion to a PS implant design. The incidence of intraoperative fracture risk during TKA varies between 0.2-4.4% [42,43]. Intraoperative fracture risk is significantly higher in PS than in CR TKAs, with a relative risk of 4.74 [42,44]. Periprosthetic fractures are devastating complications following TKA. Elkabbani et al. conducted a retrospective review in which CR TKAs demonstrated a lower risk of periprosthetic fracture compared to PS TKAs, with a risk ratio of 0.10 [45]. CR implant designs carry the advantage of avoiding additional femoral resection and iatrogenic creation of bony stress risers that risk intraoperative fracture and later periprosthetic fracture. 

Pain

Persistent pain after TKA is one of the major causes of TKA revision [37]. Pain can be idiopathic or associated with mechanical symptoms. Knee Society Pain Scores are mostly equivalent between CR and PS designs [19,20,23,24]. A systematic review and meta-analysis by Lewis et al. found that persistent post-operative knee pain was more closely associated with greater severity of pre-operative pain, psychiatric illness, and presence of other medical comorbidities rather than choice of TKA prosthetic model [46]. 

Instability

Due to preservation of the PCL, CR implants are less constrained than PS implants and theoretically less stable. Instability following TKA is clinically important with an incidence of approximately 7.5% [35] and is a significant cause of revision [36]. A retrospective review by Hannon et al. found CR design to be a risk factor for surgical revision due to instability [47]. Savov et al. reviewed patients with valgus OA who underwent either CR or PS TKA, and results showed 8% of patients in the CR group underwent revision due to instability compared to no patients in the PS group [48].

Contrarily, a systematic review by Rouquette et al. on tibiofemoral dislocation after TKA revealed dislocations were 30% CR designs and 62% PS designs [49]. Additionally, a meta-analysis of over 4,000 TKA procedures by Migliorini et al. found no differences between CR and PS designs in terms of joint instability [26]. Thus, while some literature suggests CR TKA may increase risk of instability compared to PS TKA, uncertainty remains as other studies support equivalent risks.

Intraoperative Conversion to PS

Unexpected intraoperative conversion from a CR implant design to a PS implant design is another intraoperative consideration during TKA. Iatrogenic PCL injury or ligamentous incompetency may require the additional constraint provided by PS designs. One study reports a 9% PS conversion rate of attempted CR TKAs [50]. Wang et al. report PS conversion is associated with a thicker polyethylene insert, a result of additional bony resection [51]. Severe varus deformity, flexion contracture, and narrow femurs are additional reported risk factors for conversion from CR to PS implants [50,51]. 

## Conclusions

The cruciate-retaining TKA design is widely used, both in the United States and worldwide. Currently, the benefits and drawbacks of PCL retention in TKA for primary OA is a topic of debate with no clear advantage between CR or PS designs. Studies were also highly heterogeneous in terms of how data were presented. This review offers a summary of the recent literature available on CR TKAs.

Kinematics after TKA for OA show distinct patterns between CR and PS knees, though both models demonstrate different kinematics from the native knee. CR designs encourage less femoral rollback, resulting in clinically insignificant decreased flexion. More external rotation about the medial aspect of the knee is also seen in comparison to PS designs.

Short-term functional outcomes are relatively similar between CR and PS designs, aside from ROM. Overall, the current literature and registry data suggest improved long-term survival of CR TKA designs compared to PS designs, which becomes more pronounced greater than 10 years post-operatively. As more data becomes available, continued investigation into the long-term survival of CR and PS prostheses is indicated to corroborate or reject these early findings. 

CR TKAs have the advantage of decreased incidence of intra-operative and post-operative periprosthetic fractures compared to PS designs. Post-operative pain scores are similar between CR and PS designs. A possible increased risk of instability may be associated with CR models, but the literature is conflicting. A large database study of knee dislocation rates between patients with CR and PS implants may help clarify this hypothesis. As TKA designs and techniques continue to advance, the current body of literature supports the continued use of cruciate-retaining implants.
